# Trait Emotional Intelligence as a Predictor of Adaptive Responses to Positive and Negative Affect During Adolescence

**DOI:** 10.3389/fpsyg.2018.02525

**Published:** 2018-12-11

**Authors:** Diego Gómez-Baya, Ramón Mendoza

**Affiliations:** Department of Social, Developmental and Educational Psychology, Universidad de Huelva, Huelva, Spain

**Keywords:** trait emotional intelligence, response styles, positive affect, adolescence, longitudinal

## Abstract

**Introduction and aim :** The examination of trait emotional intelligence as an important component of adolescent psychological adjustment and coping has received a great deal of attention. Trait emotional intelligence is expected to reduce the vulnerability to emotional problems by reducing mood deterioration in adverse situations. Most research to date has addressed the regulation of negative affective states, with less attention paid to the responses to positive affect. Thus, the aim of this research was to examine the cross-sectional and prospective associations between trait emotional intelligence dimensions (i.e., trait emotional attention, trait emotional clarity, and trait emotional repair), response styles to negative affect (i.e., depressive rumination and distraction) and response to positive affect (i.e., emotion-focused and self-focused positive rumination and dampening) in adolescence.

**Methods:** A 1-year follow-up study was conducted with a sample of 880 adolescents (52.4% girls) aged 14–17 years old (*M* = 14.74, *SD* = 0.68) who were enrolled in 18 high schools in Andalusia (Spain). Participants completed self-report measures of trait emotional intelligence, response to negative affect and response styles to positive affect. To analyse the data, hierarchical regression analyses and path analysis were performed.

**Results:** Our results showed that high trait emotional attention was cross-sectionally and longitudinally associated with more dampening of positive affect and more depressive rumination. Furthermore, high trait emotional repair was cross-sectionally and longitudinally related to more distraction to negative affect and more self-focused positive rumination. Some gender differences were also found; girls reported higher trait emotional attention, higher dampening, and higher depressive rumination. Furthermore, boys reported higher trait emotional repair, higher self-focused positive rumination and higher distraction to negative affect.

**Conclusions and discussion:** Our findings provide longitudinal evidence of the relationships between trait emotional intelligence and responses to both positive and negative affect during adolescence. Consequently, interventions designed to promote resilience during adolescence could target the development of more adaptive responses to both negative and positive affect within the framework of school-based emotional education programmes.

## Introduction

Over time, adolescents become more realistic in their appraisals of their own strengths and weaknesses and present a greater self-consciousness regarding peer comparison (Harter, 2015). After pubertal growth, a heightened emotional sensitivity can reduce confidence in the ability to understand and manage affects and emotions (Steinberg, 2005). In turn, adolescents have to face new and complex experiences from a position of relative inexperience and fragile emotional stability. Consequently, adolescents deal with important life dilemmas before being fully prepared, since the development of self-regulation abilities is a lengthy process. Therefore, studying the factors that favor or hinder the development of emotional self-regulation in adolescence is important to effectively promote emotional well-being during the transition to adulthood.

The literature to date has concluded that emotional intelligence (EI) is an important component of adolescent psychological adjustment, and coping has recently drawn great interest. Within EI, two constructs have been separated: trait EI and ability EI (Petrides and Furnham, 2000a). Trait EI is a cluster of emotion-related self-perceptions and dispositions located at the lower levels of personality hierarchies and is assessed using self-report measures (Pérez-González and Sanchez-Ruiz, 2014; Petrides et al., 2016). Ability EI refers to actual emotion-related abilities and is measured by maximum-performance tests (Petrides and Furnham, 2003). Thus, the instruments used for evaluating EI, self-reports or maximum-performance measures, affect the operationalization of this variable (Petrides et al., 2007b). In Belgium, Mikolajczak et al. (2009b) showed that trait EI prevents vulnerability to emotional problems by reducing mood deterioration following stressful situations. Regarding the relationship between trait and ability EI in adolescent psychological adjustment, in the UK, Davis and Humphrey (2012a) indicated that ability EI allowed a more flexible selection of coping strategies whereas trait EI increased the effectiveness of coping by strengthening the beneficial effects of active coping and reducing the detrimental consequences of avoidance. In a later work, Davis and Humphrey (2014) concluded that the effect of ability EI on adolescent adaptive coping depended on perceived competency, or trait EI. They argued that despite high scores in ability EI, low self-confidence activates avoidant coping, which does not attenuate the impact of stress. Also in the UK, Mikolajczak et al. (2009a) concluded that trait EI was associated with more adaptive coping styles, which in turn were related to fewer self-harm behaviors in adolescence. In a sample of Italian adolescents, low trait EI was determined to mediate between high-perceived parental psychological control and more frequent internalizing and externalizing problems (Gugliandolo et al., 2015). Also in Italy, Mancini et al. (2017) concluded that high trait EI is also related to improved scholastic achievement. Thus, trait EI is expected to play a key role in adolescents' psychological adjustment (Davis and Humphrey, 2012b).

To assess trait EI, research to date has provided some instruments although only a few have robust empirical validation and a theoretical framework (Pérez-González et al., 2005). One of the scales with greater empirical support is the Trait Meta-Mood Scale (TMMS; Salovey et al., 1995), which has been widely used for assessing individual differences in one's own perceptions of three emotional skills: trait emotional attention, trait emotional clarity, and trait emotional repair. Trait emotional attention refers to the attention level paid to one's own emotions, trait emotional clarity is defined as the degree of understanding of one's own emotional states, and trait emotional repair is the perceived skill to regulate one's own emotions and deal with negative emotional states. In this theoretical framework, developed by Salovey et al. (1995), a high trait EI would be composed of higher scores in trait emotional clarity and emotional repair as well as moderate punctuations in trait emotional attention. Some gender differences in trait EI have been observed in adolescence. Although boys present lower ability EI (Salguero et al., 2012), the boys believed that they had greater EI than girls (Petrides and Furnham, 2000b). Using the TMMS, Extremera et al. (2007) concluded that girls paid closer attention to their emotions whereas boys showed higher emotional clarity and emotional repair. Szymanowicz and Furnham (2013) concluded that women seem to underestimate their emotional abilities, whereas men tend to overstate them. A recent longitudinal study by Gómez-Baya et al. (2017a) concluded that lower trait emotional clarity and lower trait emotional repair in adolescent girls were associated with increased depressive symptoms.

There is some empirical evidence of the cross-sectional associations between moderate trait emotional attention and higher trait emotional clarity and repair with better psychological adjustment in adolescence (Salovey et al., 2002; Fernández-Berrocal et al., 2006). However, more longitudinal research on the mechanisms implicated in these associations is needed. The primary goal of this study was to examine the longitudinal associations between trait EI dimensions and response to both negative and positive affect in adolescents' transition to adulthood. Trait emotional attention, clarity, and repair have shown incremental predictive validity over personality traits and general intelligence to explain psychological adjustment (Fernández-Berrocal and Extremera, 2008). Thus, more research is needed to demonstrate the discriminant and predictive validity of a measure of EI in the context of other relevant variables (Izard, 2001; Bastian et al., 2005; Saklofske et al., 2007), such as coping responses. In the UK, Petrides et al. (2007a) showed that trait EI was related to measures of rumination, dysfunctional attitudes, coping, and psychological adjustment, even when personality traits were controlled. In a sample of Australian adolescents, Downey et al. (2010) determined that emotional recognition, understanding and expression were associated with engagement in problem-focused coping strategies to explain problem behaviors. In the USA, Stange et al. (2013) concluded that the interaction among low trait emotional clarity, a negative attributional style and experiencing a negative life event predicted the emergence of depressive symptoms in a 9-month longitudinal study in adolescence. In a study performed in Italy, trait EI was shown to predict emotional maladjustment in adolescence after controlling for personality traits, non-verbal cognitive ability, and gender (Andrei et al., 2014). In Spain, Fernández-Berrocal et al. (2006) observed that in adolescents, higher scores in trait emotional clarity and repair were associated with fewer depressive and anxiety symptoms after controlling for self-esteem and thought suppression. In a similar vein, Ramos et al. (2007) pointed out that those undergraduates with high trait emotional clarity and repair experienced fewer intrusive thoughts and fewer negative emotional responses after a stressful event. Also in Spain, Salguero et al. (2013) showed that high trait emotional attention and low trait emotional repair were associated with more depressive rumination, which in turn was related to more depressive symptoms. In Belgium, Mikolajczak and Luminet (2008) concluded that undergraduates with high trait EI presented greater self-efficacy to cope with and appraise a situation as challenging. Furthermore, in another study of Belgian undergraduates, Mikolajczak et al. (2008) showed that trait EI fosters the selection of adaptive responses not only in the case of stress but also in situations of fear, shame, anger, sadness, and jealousy. Moreover, they observed that trait EI also promoted the selection of adaptive responses to maintain joy. Consequently, these few studies provided some evidence that better trait EI dimensions allow the use of adaptive response to repair negative emotions and to maintain positive ones. Thus, these emotion-related dispositions regarding perception, processing, regulation, and utilization of emotional information were determined to explain an increased propensity to experience positive emotions as well as a decreased propensity to experience negative ones.

Regarding coping responses, most research to date has been focused on responses to stress and negative affect and how stress and negative affect can create a vulnerability leading to the development of emotional disorders. In this area, Response Styles Theory states that the manner in which people respond to depressive symptoms influences the severity and duration of such symptoms (Nolen-Hoeksema et al., 2008). Following this theory, two types of responses to negative affect have been proposed: depressive rumination and distraction (Treynor et al., 2003). Depressive rumination is repetitive thinking about negative affect and its causes and consequences. This response style is expected to exacerbate negative emotions by developing pessimistic thoughts, which hinder adaptive coping (Nolen-Hoeksema, 1991). Instead, distraction is presented as a more adaptive response, which entails the shift of attention away from the negative affect toward neutral or pleasant thoughts and performing some actions to relieve the current affective state. Rood et al. (2009) argued in a meta-analysis of studies with samples of children and adolescents that girls begin to ruminate more frequently than boys during adolescence, increasing the risk for future and concurrent depression. A recent longitudinal study indicated that the increase in depressive symptoms in girls was related to an increase in rumination and a decrease in distraction after a 2-year follow-up during mid-adolescence (Gómez-Baya et al., 2017c).

However, studies have focused less attention on the regulation of positive emotional states (Carl et al., 2013; Gilbert et al., 2013; Davis and Suveg, 2014; Nelis et al., 2016). Diamond and Aspinwall (2003) argued that more integration is needed regarding the analysis of emotion regulation across the life span by examining the interplay between positive and negative emotional states. According to Watson et al. (1995), affectivity presents an orthogonal structure, with negative and positive affect acting as two separate components. Thus, it is also necessary to examine the strategies that adolescents utilize in response to positive affect to better understand their psychological adjustment (Fredrickson, 2001; Bryant et al., 2011). Feldman et al. (2008) examined different responses to positive affect, i.e., dampening and two forms of positive rumination (self-focused and emotion-focused). Dampening is a response to positive affect that reduces the presence and intensity of positive affect by considering such positive emotions to be transient, by thinking about the appearance of future negative emotions or by considering that they did not deserve such positive feelings. Instead, positive rumination is expected to maintain and amplify the positive affect. Within positive rumination, emotion-focused positive rumination refers to savoring the positive emotions and their somatic sensations; self-focused positive rumination involves a self-attribution of positive affect to own positive qualities or the accomplishment of self-relevant aims. Feldman et al. (2008) showed that both higher and lower dampening and self-focused and emotion-focused positive rumination were associated with poorer adolescent adjustment, i.e., lower self-esteem and more depressive symptomatology. A recent study indicated that girls presented higher dampening and lower self-focused positive rumination than boys and that these gender differences longitudinally explained the increase in depressive symptoms in girls during middle adolescence (Gomez-Baya et al., 2017b). Other recent longitudinal work concluded that the use of adaptive responses to positive affect, specifically more self-focused positive rumination and less dampening, was interrelated with more self-esteem and life satisfaction after a one-year tracking in adolescence (Gomez-Baya et al., 2018).

### Study Justification, Aim, and Hypotheses

Most research to date has presented cross-sectional results regarding the associations between trait EI and psychological adjustment, but more analysis is required regarding explanatory coping mechanisms implications and some possible gender differences. Thus, research should further examine the relationships between trait EI dimensions (i.e., emotional attention, emotional clarity, and emotional repair) and responses to different affective experiences, especially in adolescence, as a transition period of remarkable importance for social and emotional development. There is a gap in the literature regarding the longitudinal associations between trait EI and responses to affective experiences in adolescence. Furthermore, few studies to date, as far as we know, have addressed the relationships between trait EI dimensions and responses to negative affect and stress and have paid scant attention to responses to positive affect. Consequently, further longitudinal examination is recommended to examine the relationships between trait emotional attention, trait emotional clarity and trait emotional repair and responses to positive affect. Importantly, research is needed to integrate the validity of trait EI dimensions to predict responses to both negative and positive affect during adolescence.

Thus, the aims of the present work were (a) to examine trait EI dimensions (i.e., emotional attention, emotional clarity, and emotional repair) and responses to positive and negative affect in adolescence by gender; (b) to study the cross-sectional associations between trait EI dimensions and responses to positive and negative affect in adolescence; and (c) to analyse the predictive validity of trait EI dimensions for responses to both types of affect in adolescence. Several hypotheses were developed with these goals in mind. First, girls were expected to present low trait EI (i.e., greater emotional attention and lower scores in emotional clarity and repair), consistent with Extremera et al. (2007), and fewer adaptive responses to negative affect, i.e., more depressive rumination. Girls were also expected to present less distraction and positive affect, i.e., more dampening and less positive rumination compared with boys, as reported by Rood et al. (2009) and Gomez-Baya et al. (2017b). Second, positive cross-sectional associations are expected of trait EI with distraction and positive rumination, consistent with Petrides et al. (2007a). Specifically, moderate attention and higher clarity and repair were hypothesized to be associated with more distraction and positive rumination. Negative associations were hypothesized regarding trait EI with depressive rumination and dampening, consistent with Mikolajczak et al. (2008) and Salguero et al. (2013), so that greater emotional attention and lower emotional clarity and repair would be related to more depressive rumination and dampening. Third, trait EI dimensions were expected to show predictive validity regarding responses to negative and positive affect in adolescence so that better trait EI (i.e., moderate emotional attention and higher emotional clarity and repair) would be prospectively related to more adaptive responses to both types of affects, as noted by Mikolajczak et al. (2008).

## Methods

### Participants

A sample of 880 adolescents (52.4% girls), aged between 14 and 17 years old (38.2% of the sample was 14 years old, 51.4% was 15, 8.9% was 16, and 1.6%, 17 years old) participated in this study (*M* = 14.74, *SD* = 0.68). At the beginning of the study, they were enrolled in the academic years 8 (46%) and 9 (54%) in 18 secondary schools in Andalusia, an autonomous community in southern Spain. Students who had not repeated a year were 14 and 15 years old in grades 8 and 9, respectively. Given the compulsory nature of secondary education, secondary schools constitute an excellent environment for performing a follow-up study during adolescence. The schools were sampled using convenience sampling and included secondary schools with different types of ownership (4 schools were public and 14 were private), various neighborhoods with different levels of socio-economic status (33.3% low, 33.3% medium, and 33.3% medium-high social classes) and various habitats (55.6% urban, 22.2% semi-urban, and 22.2% rural). The participating classes were randomly selected within each secondary school.

### Instrument and Variables

The self-report instrument was composed of three scales to measure trait emotional intelligence, responses to positive affect and responses to negative affect, as well as five demographic questions to assess age (“In what year were you born?,” “In what month were you born?,” “On what day were you born?”), academic year (“What grade are you in?”) and gender (“What is your gender?”).

#### Trait Emotional Intelligence

The short and adolescent version by Gómez-Baya et al. (2017a) of the Spanish adaptation of the Trait Meta-Mood Scale (TMMS; Salovey et al., 1995; Fernandez-Berrocal et al., 2004) was administered. It consists of 12 items, which are presented by the following introduction: “Read each sentence carefully and answer how often you do or think each of the things indicated.” Five Likert-type response options are presented, ranging from “never”(1) to “very often”(5). The 12 items are separated into three 4-item subscales concerning the three dimensions of trait EI: trait emotional attention or how often adolescents paid close attention to their emotional states (e.g., “I usually worry about what I feel” and “I normally spend time thinking about my emotions”); trait emotional clarity or how often adolescents perceived own emotions with clarity (e.g., “I usually know how I feel” and “I understand my feelings”); and trait emotional repair or how often adolescents succeed in repairing their negative emotional states (e.g., “Although sometimes I feel sad, I usually have an optimistic view” and “I try to have positive thoughts when I feel upset”). An overall score is calculated after adding the responses to the four items for each subscale. The scores ranged from 4 to 20 in each dimension of trait emotional intelligence; a higher score means higher trait emotional intelligence. In the validation study by Fernandez-Berrocal et al. (2004), the Spanish measure showed good reliability in each dimension (emotional attention, α = 0.90; emotional clarity, α = 0.90, and emotional repair, α = 0.86). In the two assessments of the present study, each dimension presented notable internal consistency reliability, as calculated by Cronbach's α. Specifically, trait emotional attention presented an α = 0.91 in Time 1 and α = 0.92 in Time 2. Trait emotional clarity reached an α = 0.84 in Time 1 and α = 0.87 in Time 2. With regard to trait emotional repair, notable reliability (α = 0.89 in Time 1 and α = 0.90 in Time 2) was also observed. Concerning test-retest reliability in this work, high associations were observed in emotional attention between Time 1 and Time 2, *r*_(798)_ = 0.46, *p* < 0.001, between emotional clarity in Times 1 and 2, *r*_(798)_ = 0.40, *p* < 0.001, and between the two assessments of emotional repair, *r*_(798)_ = 0.46, *p* < 0.001.

#### Responses to Positive Affect

The reduced adaptation for Spanish adolescents by Gomez-Baya et al. (2017b) of the Responses to Positive Affect Questionnaire (Feldman et al., 2008) was administered. The scale presents 12 sentences about what “someone might do when he or she feels cheerful, happy or content.” Like the original version for adult population, this adolescent adaptation is composed of three subscales: emotion-focused positive rumination (e.g., “I think about how happy I feel” and “I notice how I feel full of energy”), self-focused positive rumination (e.g., “I think, ‘I am achieving everything’” and “I think, ‘I am living up to my potential’” and dampening (e.g., “I think, ‘My streak of luck is going to end soon’” and “I think about things that could go wrong”). Four Likert-type responses were offered to assess how often the participants engaged in the actions described, ranging from “almost never” (1) to “almost always” (4). The scores were added for each dimension, and they ranged from 4 to 16; a higher score means higher frequency of the use of that response to positive affect. In the original version by Feldman et al. (2008), the dimension of emotion-focused positive rumination (α = 0.76), self-focused positive rumination (α = 0.73) and dampening (α = 0.72) presented good internal consistency. In the present research, the internal consistency was notable for all subscales: for emotion-focused positive rumination, α = 0.87 in both assessments; for self-focused positive rumination, α = 0.89 in Time 1 and α = 0.88 in Time 2; and for dampening, the internal consistency was α = 0.87 in both times. With regard to test-retest reliability in this work, emotion-focused positive rumination assessments in Times 1 and 2 were positively related, *r*_(798)_ = 0.44, *p* < 0.001, as were both measures of self-focused positive rumination, *r*_(798)_ = 0.46, *p* < 0.001. In addition, dampening in Time 1 and dampening in Time 2 were significantly correlated, *r*_(798)_ = 0.47, *p* < 0.001.

#### Responses to Negative Affect

A reduced version of the Spanish adaptation of the Children's Response Styles Scale (CRSS; Ziegert and Kistner, 2002) was used. The CRSS was adapted to Spanish adolescents by Extremera and Fernández-Berrocal (2006), and the shortened version was validated by Gómez-Baya et al. (2017c), reporting good psychometric properties. This scale was composed of 12 sentences introduced by the following explanation: “Indicate how often you do each of the following things when you feel sad or depressed.” This questionnaire is composed of two subscales with six items each. The first subscale examined depressive rumination, e.g., “I go away by myself and think about why I feel this way” and “I think, ‘Why can't I stop feeling this way?” The other subscale evaluates distraction responses, e.g., “I do something I really like to do” and “I think, ‘I'm going to do something to make myself feel better.” A four-point Likert-response scale was presented for each statement, ranging from “almost never”(1) to “almost always”(4). Overall scores were calculated for both subscales by adding the responses to their respective items. The scores ranged from 6 to 24; a higher total punctuation means a more frequent use of this kind of response to negative affect. In the validation study by Extremera and Fernández-Berrocal (2006), rumination (α = 0.89) and distraction (α = 0.80) presented notable reliability. Moreover, in the present study, both the rumination scale and the distraction scale showed notable internal consistency reliability (Rumination: Time 1, α = 0.82 and Time 2 α = 0.81; Distraction: Times 1 and 2, α = 0.86). Regarding test-retest reliability in this study, rumination measures in Times 1 and 2 were associated, *r*_(798)_ = 0.44, *p* < 0.001, as were the distraction assessments, *r*_(798)_ = 0.42, *p* < 0.001.

### Overall Study Design and Data Collection

A prospective study was performed with two evaluations separated by 1 year to allow the establishment of relationships between antecedents and consequents. The first assessment was performed in the months of April and May of 2012, and the second was conducted 1 year later. Regarding data collection in each wave, a paper-and-pencil questionnaire was anonymously and individually administered to the sample of students in each classroom of the respective high school. Further information is described elsewhere (Gómez-Baya, 2014). To allow tracking and to maintain anonymity, a code was created with the number of educational centers (1–18), birth date (day, month, and year), and gender (1-boy, 2-girl). No student refused to participate in the study. Neither the students nor the schools received any type of compensation. All principles in the Helsinki Declaration were respected, and written informed consent was signed by all adolescents and their parents. This research obtained formal approval from the University of [Anonymised]'s ethics committee.

### Data Analysis Design

To examine the normality of the variables, the Kolmogorov-Smirnov test was conducted. Although this test showed that all variables were non-normally distributed (*p* < 0.001), a large sample size is robust to normality violations, according to Altman and Bland (1995). Consequently, parametric statistical tests were performed, using an α level of 0.05. Furthermore, missing values were below 5% in each separate question in the overall study. Little's test indicated that missing values were distributed completely randomly, $\chi_{\left(782,\text{N}\;=\;880\right)}^{2}$ = 837.39, *p* = 0.083. Then, an expectation-maximization imputation procedure was conducted following the indications by Gold and Bentler (2000).

First, descriptive statistics were examined, i.e., mean and standard deviation, and differences by gender and age in all variables were examined by conducting a multivariate variance analysis. Second, Pearson zero-order bivariate correlations were calculated among study variables in Times 1 and 2. Third, five stepwise regression analyses were conducted to examine the predictive validity of trait emotional intelligence dimensions in Time 1 for each response to positive (i.e., emotion-focused positive rumination, self-focused positive rumination, and dampening) and negative affect (i.e., depressive rumination and distraction) in Time 2. In the first step of these analyses, gender and age were introduced; in the second, each response to positive and negative affect in Time 1 was added. Thus, after controlling for demographics and initial values in each response, in the third step, the three dimensions of trait emotional intelligence in Time 1 were introduced (i.e., trait emotional attention, trait emotional clarity, and trait emotional repair) to explain scores for each response after a 1-year follow-up. These analyses were carried out with the statistical package SPSS 21.0 (IBM Corp, 2012).

Fourth, a path analysis model was performed to integrate the results from previous regression analysis within a confirmatory model. This model tested (a) the bidirectional relationships between dimensions of trait emotional intelligence and responses to positive and negative affect in Time 1 and (b) the relationships between trait emotional intelligence's dimensions and responses in Time 1 (as antecedents) and responses in Time 2 (as consequents). Thus, this model tested the incremental predictive validity of trait emotional intelligence for responses to positive and negative affect after controlling for the initial values in these responses. This structural equation model was tested with program EQS 6.1 (Byrne, 2013). The effects described in this model only represent causal assumptions because there is no variable manipulation (Bollen and Pearl, 2013). Measurement equations were examined by analyzing standardized coefficients to estimate the effect of variables. To examine the overall model fit, the χ^2^ test, the Bentler comparative fit index (CFI), the root mean square error of approximation (RMSEA), and a 90% confidence interval of RMSEA were calculated, following the instructions of Hu and Bentler (1999) and Jöreskog (1993). In addition, standardized residuals were analyzed.

## Results

### Descriptive Statistics and Bivariate Correlations

Table 1 presents the mean and standard deviation of study variables in Times 1 and 2 as well as Pearson bivariate correlations. Moderate mean scores were observed in the dimensions of trait emotional intelligence in Times 1 and 2, with the lower punctuations in trait emotional attention. Regarding responses to positive affect, emotion-focused positive rumination presented the highest mean scores while dampening was lowest in Times 1 and 2. Concerning responses to negative affect, distraction responses were more frequently used on average than depressive rumination.

**Table 1:** Descriptive statistics and Pearson zero-order bivariate correlations of study variables in Times 1 and 2.

	***M***	***M*_**boys**_(*SD*)**	***M*_**girls**_(*SD*)**	**1**	**2**	**3**	**4**	**5**	**6**	**7**	**8**	**9**	**10**	**11**	**12**	**13**	**14**	**15**	**16**
1.AT1	13.24(4.07)	12.37(4.21)	14.04(3.78)	1															
2.AT2	13.19(4.01)	12.50(4.08)	13.81(3.85)	0.46^***^	1														
3.CL1	13.79(3.54)	13.87(3.72)	13.71(3.36)	0.23^***^	0.14^***^	1													
4.CL2	13.70(3.52)	14.11(3.64)	13.33(3.36)	0.06	0.18^***^	0.40^***^	1												
5.RE1	13.59(4.22)	13.96(4.18)	13.24(4.22)	0.12^**^	0.03	0.45^***^	0.21^***^	1											
6.RE2	13.46(4.12)	14.00(4.09)	12.97(4.09)	0.05	0.08^*^	0.21^***^	0.35^***^	0.46^***^	1										
7.EF1	12.46(3.11)	12.59(2.95)	12.35(3.25)	0.26^***^	0.15^***^	0.35^***^	0.19^***^	0.40^***^	0.23^***^	1									
8.EF2	12.28(3.10)	12.26(3.05)	12.29(3.14)	0.19^***^	0.26^***^	0.19^***^	0.28^***^	0.19^***^	0.32^***^	0.44^***^	1								
9.SF1	10.55(3.48)	10.91(3.33)	10.22(3.57)	0.16^***^	0.06	0.36^***^	0.20^***^	0.37^***^	0.30^***^	0.55^***^	0.30^***^	1							
10.SF2	10.26(3.41)	10.58(3.21)	9.97(3.56)	0.12^**^	0.17^***^	0.20^***^	0.31^***^	0.24^***^	0.39^***^	0.37^***^	0.53^***^	0.46^***^	1						
11.DA1	7.85(3.44)	7.78(3.50)	7.91(3.38)	0.20^***^	0.09^***^	−0.11^***^	−0.07^*^	−0.17^***^	−0.16^***^	−0.01	−0.01	−0.10^***^	0.01	1					
12.DA2	7.68(3.30)	7.32(3.19)	8.00(3.36)	0.22^***^	0.22^***^	−0.04	−0.11^***^	−0.04	−0.19^***^	−0.03	−0.06	−0.12^***^	−0.06	0.47^***^	1				
13.RU1	15.76(4.47)	14.90(4.57)	16.55(4.24)	0.55^***^	0.32^***^	0.10*^*^	0.03	−0.02	−0.07^*^	0.17^***^	0.12^***^	0.01	0.02	0.25^***^	0.19^***^	1			
14.RU2	15.96(4.35)	15.13(4.38)	16.71(4.18)	0.31^***^	0.51^***^	0.05	0.04	−0.01	−0.07^*^	0.12^***^	0.21^***^	−0.01	0.09*^*^	0.15^***^	0.25^***^	0.44^***^	1		
15.DI1	16.19(4.76)	16.86(4.40)	15.58(4.98)	0.05	−0.04	0.31^***^	0.21^***^	0.59^***^	0.33^***^	0.30^***^	0.16^***^	0.31^***^	0.20^***^	−0.03	0.02	−0.03	−0.07^*^	1
16.DI2	16.04(4.66)	16.66(4.43)	15.48(4.80)	0.01	−0.01	0.22^***^	0.23^***^	0.36^***^	0.51^***^	0.18^***^	0.27^***^	0.21^***^	0.33^***^	0.01	−0.02	−0.09*^*^	−0.05	0.42^***^	1

*AT, trait emotional attention; CL, trait emotional clarity; RE, trait emotional repair; EF, emotion-focused positive rumination; SF, self-focused positive rumination; DA, dampening; RU, depressive rumination; DI, distraction; 1, time 1; 2, time 2*.

* *p < 0.05*,

** *p < 0.01*,

*** *p < 0.001*.

Furthermore, correlations analysis indicated significant associations among study variables. First, trait emotional attention presented small positive relationships with responses to positive affect while moderate positive relationships were observed with depressive rumination. Second, trait emotional clarity and trait emotional repair presented small to moderate positive associations with self-focused and emotion-focused positive rumination and distraction and small negative correlations with dampening. Third, scores on the variables in Time 1 showed moderate to high positive correlations in the scores 1 year later. Fourth, moderate positive associations were observed between trait emotional clarity and trait emotional repair whereas trait emotional attention showed small positive associations with those dimensions at each assessment time. Fifth, some associations may be consistently described between the responses in both waves: moderate positive associations were observed between emotion-focused and self-focused positive rumination dimensions; depressive rumination presented small positive associations with emotion-focused positive rumination and dampening; and distraction showed small positive associations with both types of positive rumination.

Table 1 also presents the descriptive statistics by gender of study variables in Times 1 and 2. Multivariate variance analysis showed some gender differences. Girls reported higher trait emotional attention in Time 1, *F*_(1, 878)_ = 37.34, *p* < 0.001, and Time 2, *F*_(1, 878)_ = 23.35, *p* < 0.001. Boys showed more trait emotional repair in Time 1, *F*_(1, 878)_ = 6.09, *p* = 0.014, and Time 2, *F*_(1, 878)_ = 14.36, *p* < 0.001, and more trait emotional clarity in Time 2, *F*_(1, 878)_ = 11.30, *p* = 0.001. Concerning responses to positive and negative affect, boys reported more self-focused positive rumination in Time 1, *F*_(1, 878)_ = 4.19, *p* = 0.006, and Time 2, *F*_(1, 878)_ = 2.96, *p* = 0.032, as well as more distraction in Time 1, *F*_(1, 878)_ = 7.15, *p* < 0.001, and Time 2, *F*_(1, 878)_ = 5.02, *p* = 0.002. Girls showed more depressive rumination in Time 1, *F*_(1, 878)_ = 10.72, *p* < 0.001, and Time 2, *F*_(1, 878)_ = 11.56, *p* = 0.002, and more dampening in Time 2, *F*_(1, 878)_ = 4.05, *p* = 0.007. Furthermore, no substantial differences were observed by age. Only significant differences were presented in dampening in Time 1, *F*_(3, 876)_ = 3.69, *p* = 0.012, and trait emotional attention in Time 2, *F*_(3, 876)_ = 4.17, *p* = 0.006, with higher scores in older adolescents.

### Stepwise Regression Analyses

Table 2 describes the results from stepwise regression analyses for responses to positive affect as criteria variables in Time 2. These analyses showed that the best predictors of each response to positive affect in Time 2 were the initial level of the same response 1 year before. After controlling for the initial levels of each response and demographics (i.e., gender and age), the results indicated that trait emotional repair in Time 1 was positively related to self-focused positive rumination in Time 2 while trait emotional attention in Time 1 showed a positive relationship with dampening after the follow-up. No relationship was observed between trait emotional intelligence in Time 1 and emotion-focused positive rumination in Time 2. Explained variance was above 20% for self-focused positive rumination and dampening. Although the majority of the explained variance accounted for the initial values in these responses, the increases in *R*^2^ after adding trait emotional intelligence dimensions were significant in both self-focused positive rumination, Δ*R*^2^ = 0.01, Δ*F*_(3, 873)_ = 3.29, *p* = 0.020, and dampening, Δ*R*^2^ = 0.01, Δ*F*_(3, 873)_ = 5.05, *p* = 0.002.

**Table 2:** Stepwise regression analyses for responses to positive affect as criteria variables.

**Criteria 2**	**Emotion-focused positive rumination**				**Self-focused positive rumination**				**Dampening**			
	***R*^**2**^**	***F***	***t***	**β**	***R*^**2**^**	***F***	***t***	**β**	***R*^**2**^**	***F***	***t***	**β**
1st Step	0.02	3.08^*^			0.01	3.90^*^			0.02	6.82^**^		
Gender			0.42	0.02			−2.60	−0.09^*^			3.23	0.11^**^
Age			1.37	0.09			0.86	0.03			1.98	0.07^*^
2nd Step	0.20	32.13^***^			0.22	82.33^***^			0.23	86.42^***^		
Gender			0.94	0.04			−1.32	−0.04			3.26	0.10^**^
Age			0.96	0.06			2.06	0.06			0.76	0.02
Criteria 1			10.83	0.43^***^			15.40	0.46^***^			15.55	0.46^***^
3rd Step	0.20	18.75^***^			0.23	43.13^***^			0.24	46.33^***^		
Gender			0.58	0.02			−1.55	−0.05			2.50	0.08^*^
Age			0.98	0.06			2.05	0.06			0.72	0.02
Criteria 1			9.30	0.42^***^			12.86	0.43^***^			14.35	0.44^***^
AT1			1.54	0.06			1.77	0.06			3.61	0.12^***^
CL1			−0.77	−0.03			−0.19	−0.01			−0.83	−0.03
RE1			0.01	0.01			2.34	0.08^*^			1.08	0.04

*AT, trait emotional attention; CL, trait emotional clarity; RE, trait emotional repair; 1, Time 1; 2, Time 2*.

* *p < 0.05*,

** *p < 0.01*,

*** *p < 0.001*.

Table 3 shows the results of stepwise regression analyses for responses to negative affect in Time 2 as criteria variables. The best predictors of depressive rumination and distraction in Time 2 were, respectively, depressive rumination and distraction in Time 1. After controlling for these initial values and demographics (i.e., gender and age), the results showed that trait emotional attention in Time 1 had a positive relationship with depressive rumination in Time 2. Moreover, trait emotional repair was positively related to distraction 1 year later. To explain depressive rumination and distraction in these analyses, explained variance was 21%. However, the majority of the explained variance accounted for initial values in those responses to negative affect. Concerning distraction, the increase in explained variance after including trait emotional intelligence dimensions in the equation was significant, Δ*R*^2^ = 0.03, Δ*F*_(3, 873)_ = 7.56, *p* < 0.001. No significant increase was observed for rumination's explained variance, Δ*R*^2^ = 0.01, Δ*F*_(3, 873)_ = 1.84, *p* = 0.139, despite the significant effect by emotional attention in Time 1.

**Table 3:** Stepwise regression analyses for responses to negative affect as criteria variables.

**Criteria 2**	**Depressive rumination**				**Distraction**			
	***R*^**2**^**	***F***	***t***	**β**	***R*^**2**^**	***F***	***t***	**β**
1st Step	0.03	15.16^**^			0.02	7.36^**^		
Gender			5.50	0.18^**^			−3.72	−0.13^**^
Age			0.52	0.02			0.70	0.02
2nd Step	0.20	75.32^**^			0.18	65.81^**^		
Gender			3.42	0.11^**^			−2.24	−0.07^*^
Age			0.17	0.01			0.590	0.03
Criteria 1			13.75	0.42^**^			13.41	0.41^**^
3rd Step	0.21	38.68^**^			0.21	37.43^**^		
Gender			3.07	0.10^**^			−2.00	−0.06
Age			0.09	0.01			1.21	0.04
Criteria 1			10.38	0.38^**^			8.28	0.31^**^
AT1			2.31	0.09^*^			−0.92	−0.03
CL1			−0.26	−0.01			1.71	0.06
RE1			0.13	0.01			3.63	0.15^**^

*AT, trait emotional attention; CL, trait emotional clarity; RE, trait emotional repair; 1, Time 1; 2, Time 2*.

* *p < 0.05*,

** *p < 0.01*,

*** *p < 0.001*.

### Path Analysis

**Figure 1:**
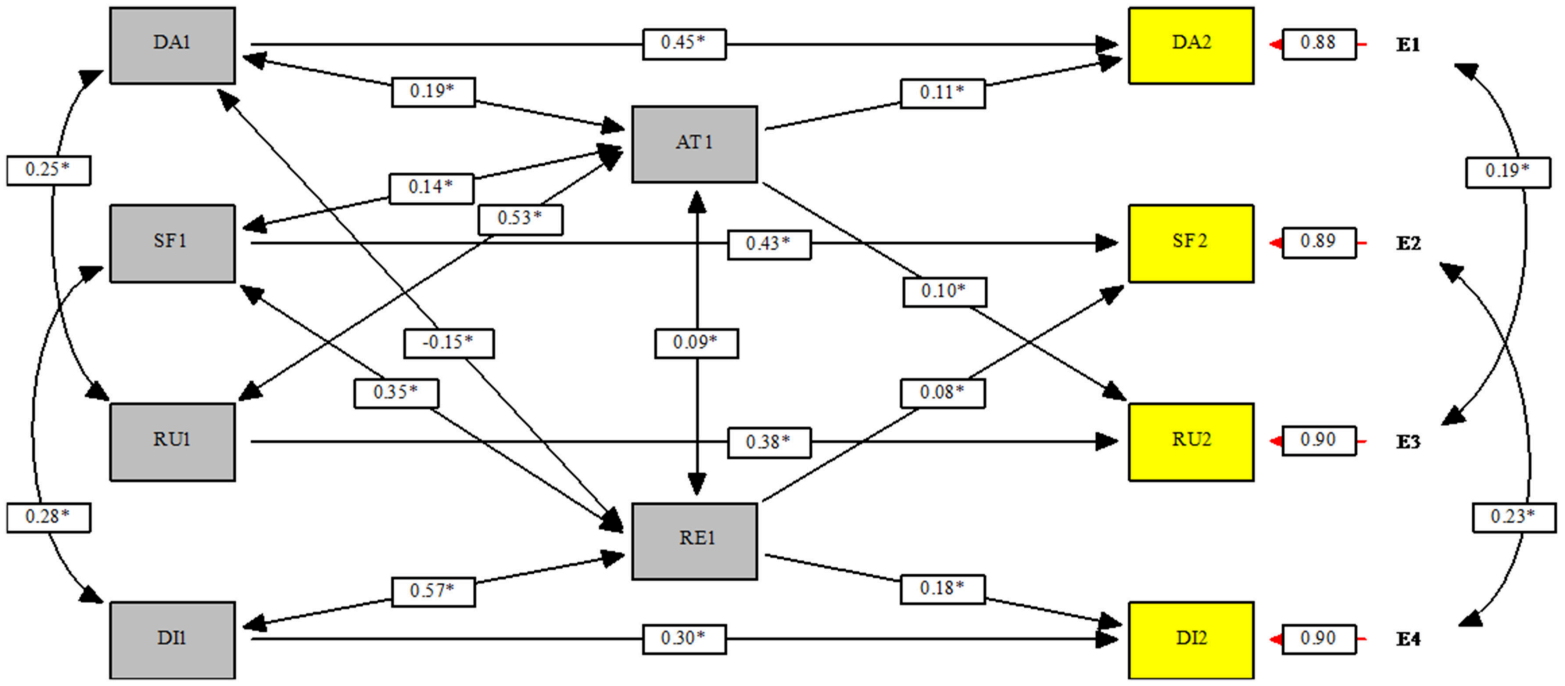
Path analysis of responses of the relationships between responses to positive and negative affects in Times 1 and 2 and trait emotional intelligence dimensions in Time 1. AT, Trait Emotional Attention; RE, Trait Emotional Repair; SF, Self-focused Positive Rumination; DA, Dampening; RU,Depressive Rumination; DI, Distraction; 1, Time 1; 2, Time 2.

Based on previous results from correlations and stepwise regression analyses, a structural equation model was tested. Because stepwise regression analysis did not show a significant longitudinal relationship between trait emotional intelligence and emotion-focused positive rumination, this response to positive affect was not included in this model. Moreover, since trait emotional clarity was not determined to be longitudinally related to responses to positive affect or to responses to negative affect, this dimension was not included in the model. Thus, the model proposed (a) the relationships between initial scores in responses to positive affect (i.e., dampening and self-focused positive rumination) and in responses to negative affect (i.e., depressive rumination and distraction) and the scores for these respective responses after the follow-up; (b) a bidirectional association between trait emotional attention and trait emotional repair in Time 1; (c) the relationships between trait emotional attention in Time 1 and dampening and depressive rumination in Time 2 and the relationships between trait emotional repair in Time 1 and self-focused positive rumination and distraction in Time 2; (d) the bidirectional associations between dampening and depressive rumination and between self-focused positive rumination and distraction in each assessment time; and (e) some bidirectional associations in Time 1 between trait emotional intelligence and responses, i.e., the association between trait emotional attention and dampening, self-focused positive rumination and depressive rumination, and between trait emotional repair and dampening, self-focused positive rumination and distraction. Figure 1 presents the path analysis indicating standardized solutions. This model reached a good overall data fit, $\chi_{\left(26,\text{N}=880\right)}^{2}$ = 65.88, *p* < 0.001, χ^2^ / df = 2.53, CFI = 0.971, RMSEA = 0.045, 90% CI RMSEA = 0.032–0.059.

All measurement equations were significant, and standardized residuals were quite low (between −0.1 and 0.1). The best predictors of responses in Time 2 were the same responses in Time 1, with all standardized solutions positive and above 0.30. The strongest relationship was observed for dampening (β = 0.45) while the lowest was observed for distraction (β = 0.30). Furthermore, the examined effects of trait emotional intelligence on responses after the follow-up were also positive and significant. Specifically, trait emotional attention in Time 1 presented similar positive effects on dampening (β = 0.11) and depressive rumination (β = 0.10). More differences were observed in the effects found between trait emotional repair in Time 1 and responses in Time 2. Thus, a greater positive effect was observed for distraction (β = 0.18) than for self-focused-positive rumination (β = 0.08). Finally, the associations in Time 1 between trait emotional attention and repair with responses also presented some interesting findings. Trait emotional attention was positively interrelated with dampening, self-focused positive rumination and depressive rumination. The strongest association was observed with regard to depressive rumination (β = 0.53) while the lowest was for self-focused positive rumination (β = 0.14). Moreover, trait emotional repair showed positive interrelations with self-focused positive rumination and distraction and a negative interrelation with dampening. The strongest association was presented for distraction (β = 0.57) and the lowest for dampening (β = −0.15). Finally, a small positive association was observed between trait emotional intelligence dimensions, and moderate positive associations at each time were observed between dampening and depressive rumination and between self-focused positive rumination and distraction.

Concerning explained variance for responses to positive affect after the follow-up, the results indicated that dampening reached a *R*^2^ = 0.23 while self-focused positive rumination showed a *R*^2^ = 0.21. With regard to responses to negative affect, depressive rumination presented a *R*^2^ = 0.20 while distraction presented a *R*^2^ = 0.19.

## Discussion

This study sought to examine trait EI dimensions (i.e., emotional attention, emotional clarity, and emotional repair) and responses to positive and negative affects in adolescence by gender and to analyse the cross-sectional and longitudinal relationships between trait EI dimensions and both types of responses. Some contributions may be described. Concerning the first goal, the results indicated that girls reported lower trait EI, i.e., increased emotional attention and lower emotional clarity and repair, consistent with results by Extremera et al. (2007) and Szymanowicz and Furnham (2013), who argued that girls seem to underestimate their emotional skills. In addition, girls indicated fewer adaptive responses to positive affect (i.e., more dampening and less self-focused positive rumination) and fewer adaptive responses to negative affect (i.e., more depressive rumination and less distraction) than boys, which is consistent with previous studies (Rood et al., 2009; Gomez-Baya et al., 2017b). These results are also consistent with our hypotheses for the first goal. Regarding the second goal, some cross-sectional associations were observed between trait EI dimensions and responses. Trait emotional attention showed positive associations with dampening, positive rumination, and depressive rumination. Furthermore, both trait emotional clarity and repair presented positive associations with distraction and positive rumination and a negative association with dampening. Thus, greater trait emotional attention appears to be associated with fewer adaptive responses while trait emotional clarity and repair are associated with more adaptive responses. No substantial differences were observed in these associations between boys and girls. The results concerning association with responses to negative affect are consistent with previous literature (Petrides et al., 2007a; Salguero et al., 2013) and with our hypotheses. The associations with responses to positive affect are an important contribution of the present work; the associations are consistent with previous research (Mikolajczak et al., 2008) and with our initial hypothesis. Finally, regarding the third aim, a confirmatory model has integrated the prospective relationships between trait EI dimensions and responses to positive and negative affect after a 1-year follow-up. After controlling for initial levels in these responses, this model showed that (a) greater trait emotional attention predicted fewer adaptive responses, i.e., high depressive rumination and high dampening, and (b) greater trait emotional repair predicted more adaptive responses, i.e., high distraction and high self-focused positive rumination. This model presenting the relationships between antecedents and consequents is the primary contribution of the present research to previous literature by integrating the predictive value of trait EI dimensions of responses to both negative and positive affect.

Some explanations may be proposed to explain the prospective association observed between trait emotional attention and maladaptive responses to positive and negative affect. Studies have shown the detrimental effects of psychological adjustment of self-focused attention. A meta-analysis by Mor and Winquist (2002) showed the general association between self-focused attention on negative aspects and events and experiencing negative emotions. However, those authors distinguished between ruminative self-focus on negative aspects and events from self-focus on positive aspects, which was determined to be related to low negative affect. In this vein, Watkins E (2004) separated adaptive and maladaptive ruminative self-focus during emotional processing. Thus, to avoid the detrimental effects of high attention to one's own emotions, the focus may be moved toward developing the ability to observe, describe and accept the present moment, non-judgementally and non-reactively, to be able to participate with full awareness in the ongoing coping activity. The cultivation of this adaptive form of self-focused attention, as in mindfulness intervention, was related to less depressive rumination and reduced emotional avoidance, which in turn improves behavioral self-regulation (Baer, 2009). Furthermore, the perceived ability to repair emotional states, or trait emotional repair, is prospectively associated with the selection of adaptive responses to both negative and positive affective states, as previously noted by Mikolajczak et al. (2008). Thus, it seems that trait emotional repair would be especially associated with the choice of adaptive response to downregulate negative emotions using distraction but also to maintain positive responses by performing self-focused positive rumination. The upregulation of positive emotions is an important consequence of emotional repair as well, which would allow for maintaining and amplifying these experiences (Livingstone and Srivastava, 2012) and preventing emotional symptoms (Fussner et al., 2015; Gomez-Baya et al., 2017b; Nelis et al., 2018). In this sense, as a consequence of the high self-efficacy in emotional repair, self-focused positive rumination would allow the attribution of the positive affect to good self-qualities, which is particularly relevant during adolescence, a life period during which self-concept becomes more complex and includes more abstract descriptions (Harter, 2015).

Thus, this work presents some important contributions to previous literature. This research has provided longitudinal evidence for the coping mechanisms associated with trait EI in adolescence. Moreover, the integrated analysis of the relationships between trait EI dimensions and responses to both positive and negative affect provided a novel result. Despite these contributions, some limitations should be acknowledged. The longitudinal study design allows for establishing associations between antecedents and consequents, but no causal inferences can be concluded because an experimental design would be required. Petrides and Furnham (2003) provided construct validity for trait EI after conducting experiments of emotion recognition and reactivity to mood induction. In this area, Matthews et al. (2015) showed that high trait EI was related to reduced stress response after an experimental task. Additional assessments or waves could be recommended in a longitudinal study to explore change trajectories during adolescence. Concerning the instrument, only self-reports were used, providing subjective information. A future line of research could come from collecting data from other relevant informants, such as parents, peers or teachers, consistent with conclusions by Boyatzis et al. (2015). Furthermore, low test-retest reliability coefficients were observed, what may be due to the 1-year interval between the two assessments and the possible changes in EI perceptions during this period in mid-adolescence, consistent with Keefer et al. (2013). These changes may be explained by the heightened emotional sensitivity (Forbes and Dahl, 2010) and the increased skill to produce more realistic self-appraisals (Harter, 2015). Finally, regarding sample composition, since a convenient procedure was used, the results cannot be generalized to Spanish adolescents. Although the sample size is remarkable, it comes from a single region in Spain, and the schools selected were not homogeneously distributed according to public or private ownership. An interesting area of future research could come from the examination of cultural differences in the associations between trait EI and coping by comparing samples from different countries. Some cultural differences in trait EI have been reported between Western individualist societies and Eastern collectivist societies (Gökçen et al., 2014) while the present work has provided some evidence from Southern Europe.

Some practical implications may be suggested from the study contributions. Schools may represent an excellent setting for implementing interventions during adolescence to promote EI and adaptive response because schools present appropriate conditions for programme implementation. The need to implement educational policies to promote emotional education has been strongly defended (Mayer and Cobb, 2000). A recent meta-analysis showed the effectiveness of universal intervention programmes with children and adolescents from the framework of the Collaborative for Academic, Social and Emotional Learning (CASEL) in developing social and emotional skills and improving psychological adjustment and academic performance (Durlak et al., 2011). Another experience of school-based intervention in EI is RULER, which was designed to improve classroom social interactions and develop emotional literacy (Brackett et al., 2012). In Spain, the INTEMO programme has provided supportive evidence for developing skills regarding emotional perception, facilitation, understanding, and management, which were effective in promoting adolescent mental health (Ruiz-Aranda et al., 2012). Furthermore, resilience education has recently emerged to develop psychological strengths and coping resources to promote resilience within the school context (Gillham et al., 2013). Our conclusions regarding the associations between EI and responses to affect underline the need to integrate the interventions from emotional education and resilience education in adolescence. Moreover, our study suggests that resilience education should also promote adaptive responses to positive affect. Most interventions have targeted the development of coping resources to face negative affect, such as the Penn Resilience Program (Gillham et al., 2007). However, school-based interventions could be improved by including activities to increase positive emotions. In this vein, Quoidbach et al. (2015) concluded that exercising, socializing and being in nature were effective in enhancing positive affect. In addition, imagining future positive events seems to present more long-term benefits to prolong positive affective states. Consequently, the prospective associations between trait EI and responses to positive and negative affect in adolescents could invite the integration of school-based interventions. Moreover, given the gender differences observed, more gender-specific activities should be designed to improve interventions' overall effectiveness, as indicated by Zeidner et al. (2002). Thus, intervention programmes should be focused on improving emotional self-efficacy beliefs in girls and their coping resources to face the experiencing of both negative and positive affective states.

## Ethics Statement

All procedures performed respected the ethical standards of the institutional research committee and Helsinki declaration. Written informed consent was obtained from all individual participants and their parents.

## Author Contributions

All authors listed have made a substantial, direct and intellectual contribution to the work, and approved it for publication.

### Conflict of Interest Statement

The authors declare that the research was conducted in the absence of any commercial or financial relationships that could be construed as a potential conflict of interest.
